# Supplementary material for: Artificial intelligence in deep brain stimulation for movement disorders: a systematic review and technology readiness assessment
Source: NPJ Digit Med. 2026 Jul 16;9:867. doi: 10.1038/s41746-026-03015-4 (PMC13745585; doi:10.1038/s41746-026-03015-4)
Supplement: Supplementary file 1 [file 41746_2026_3015_MOESM1_ESM.pdf]

Supplementary table 1: External validation studies

Author, Year	DBS Workflow Stage	Context	Internal Validation	External Validation	Robustness
Boutet et al., 2021 [1]	Programming	fMRI BOLD response patterns from PD patients with STN-DBS and GPi-DBS. LDA classifier trained on 39 a priori clinically optimized patients (35 STN, 4 GPi) to predict optimal vs. non-optimal DBS contact and voltage settings using 16 ROI features.	**Training accuracy (5-fold CV):** Combined contact + voltage model: Accuracy = 88% (n = 39) Motor regions only: Accuracy= 67% Excluding GPi patients: Accuracy = 81% Contact-only model: Accuracy = 86%	**Test Dataset 1** (a priori clinically optimized, n = 9): Combined model: Accuracy = 88% Contact-only: Accuracy= 63% Voltage-only: Accuracy = 71% **Test Dataset 2** (stimulation-naïve, n = 9): Combined model: Accuracy = 76%	**Variable.** **Dataset 1:** No performance drop, combined model maintained 88% accuracy, identical to training. **Dataset 2:** Accuracy dropped from 88% to 76% (12 percentage point decrease). Authors note this validates the model for patients not yet optimized, though the drop suggests some generalization limitation. The authors acknowledge: "generalizable ML models require large pools of data to avoid overfitting."
Boutet et al., 2021 [2]	Programming	STN-DBS for Parkinson's Disease. SVM classification of sign-specific clinical outcomes (tremor, rigidity, bradykinesia, axial signs) into quartiles of efficacy using VTA overlap with hot and cold spots. Internal cohort: TWH (n= 275); External cohort: CUB (n= 51).	**SVM Accuracy (TWH, Hot & Cold Spot Overlap):** Tremor: 240/242 (99%) Rigidity: 252/273 (92%) Bradykinesia: 275/275 (100%) Axial signs: 268/ 274 (98%)<br>**Hot Spot Only:** Tremor: 96%, Rigidity: 68%, Bradykinesia: 98%, Axial signs: 88% **Cold Spot Only:** Tremor: 41%, Rigidity: 53%, Bradykinesia: 72%, Axial signs: 58% **Voltage Only:** Tremor: 48%, Rigidity: 45%, Bradykinesia: 44%, Axial signs: 44%	**SVM Accuracy (CUB, Hot & Cold Spot Overlap):** Tremor: 40/40 (100%) Rigidity: 46/48 (96%) Bradykinesia: 51/51 (100%) Axial signs: 50/50 (100%) **Hot Spot Only:** Tremor: 90%, Rigidity: 79%, Bradykinesia: 98%, Axial signs: 84% **Cold Spot Only:** Tremor: 80%, Rigidity: 71%, Bradykinesia: 94%, Axial signs: 76% **Voltage Only:** Tremor: 55%, Rigidity: 58%, Bradykinesia: 55%, Axial signs: 60%	**High resilience.** External validation accuracy was equal to or higher than internal validation for the combined hot & cold spot model: Tremor: 99% → 100% (+1%) Rigidity: 92% → 96% (+4%) Bradykinesia: 100% → 100% (no change) Axial signs: 98% → 100% (+2%) The authors state: "although the position of active contact differed slightly between institutions, the external validation maintained a high accuracy, emphasizing the robustness of our model." Model demonstrated robust generalization across institutions with no performance degradation.
Shamir et al., 2019 [3]	Targeting	ML method trained on 7T MRI database of 46 PD patients (7T-ML) to predict STN shape and	Previously validated against same-patient 7T MRI (Kim et al., 2015, ref 43). Specific	STN trajectory length: 7T-ML = 5.8 ± 0.9 mm vs MER = 6.2 ± 0.7 mm ($P > .05$). Correlation: r = 0.41, P <	**High Resilience.** Model trained at University of Minnesota, tested on independent cohort at Hadassah Medical Center, Jerusalem

		position on clinical 3T MRI for STN-DBS targeting. Validated against intraoperative MER in 16 PD patients (30 implanted leads) at an independent center.	internal validation metrics not reported in this paper.	.01. Contact location classification agreement: 93% (112/120). Average absolute difference in STN lengths: 0.75 mm (SD = 0.59 mm). All 30 trajectories intersecting STN per MER also intersected per 7T-ML.	(blinded). Average absolute STN length difference: 0.75 mm (SD = 0.59 mm). 8/120 contacts (7%) misclassified, all at dorsal STN border. Regression slope = 0.52 (ideal = 1), attributed to measurement error. 93% cross-site agreement on contact classification despite real-life intraoperative error sources. Authors note strong correspondence between methods.
Valsky et al., 2020 [4]	Targeting	Striato-pallidal border classification (Str-GPe, GPe-GPi, GPi-exit) from MER background spiking activity in GPi-DBS for Parkinson's disease and dystonia (awake and lightly anesthetized). HMM-based algorithm using NRMS and power spectra features.	Leave-one-trajectory-out cross-validation (116 trajectories, 42 patients, single center):<br>PD Str-GPe: 84% accuracy within 0.3 mm PD<br>GPe-GPi: 78% accuracy within 0.3 mm PD GPi-exit: 95% accuracy within 0.3 mm	Prospective test on 27 trajectories, 10 patients, 3 medical centers (Hadassah, Sourasky, Rabin):<br>PD Str-GPe: 95% accuracy within 0.3 mm<br>PD GPe–GPi: 45% accuracy within 0.3 mm<br>PD GPi-exit: 83% accuracy within 0.3 mm	**Variable by anatomical clarity:**<br>Str-GPe: Improved from 84% to 95% on external test. GPe–GPi: Dropped from 78% to 45% (33 pp drop); expert uncertain in 47% of training trajectories. GPi-exit: Dropped from 95% to 83% (12 pp drop).
Chang et al., 2025 [5]	Patient Selection / Outcome Prediction	Comprehensive clinical scale data (demographics, MMSE, HAMA, UPDRS-III, PDSS, etc.) from Parkinson's Disease patients undergoing bilateral STN-DBS; LightGBM model predicting motor symptom improvement at 1 year post-DBS	AUC = 0.874 (95% CI [0.822–0.927]); 5-fold cross-validation average AUC = 0.921 ± 0.03; Accuracy = 83.1%; F1 score = 57.1%; Recall = 52.4%; Precision = 62.9%; Balanced accuracy = 0.812; F1 macro = 0.801	AUC = 0.769 (95% CI [0.685 - 0.853]); Balanced accuracy = 0.753; F1 macro = 0.742 (External validation on 123 patients from Qingdao Municipal Hospital)	**Moderate drop.**<br>AUC dropped from 0.874 (internal) to 0.769 (external), a decrease of 0.105.<br>Balanced accuracy dropped from 0.812 to 0.753 (decrease of 0.059). F1 macro dropped from 0.801 to 0.742 (decrease of 0.059).<br>Authors state: "The slightly lower AUC in the external validation set may be attributed to differences in patient demographics, clinical practices, or data collection protocols between the two centers." Performance described as "robust generalizability" and "clinically meaningful."

Habets et al., 2022 [6]	Patient selection / outcome prediction	Preoperative clinical variables from Parkinson's Disease patients undergoing STN DBS.<br>**Model:** DBS-PREDICT (logistic regression), predicting weak vs. strong motor responders 1 year post-surgery.<br>**Variables:** Age at PD onset, age at DBS, disease duration, gender, LEDD, UPDRS II, III (on/off-med), IV, HY (on/off-med).<br>**Validation cohort:** 322 PD patients from 6 international centers (Europe, Asia, North America).	**Development study (single-center, n=90, 10-fold cross-validation):**<br>AUC = 0.88 (±0.14)<br>Accuracy = 78%	**External multicenter validation (n=322):**<br>AUC = 0.76<br><br>**Threshold 0.50:**<br>Accuracy = 0.77 (248/322)<br>Sensitivity = 0.29 (24/83)<br>Specificity = 0.94 (224/239)<br>PPV = 0.62 (24/39)<br>NPV = 0.79 (224/283)<br><br>**Threshold 0.24:**<br>Accuracy = 0.73 (236/322)<br>Sensitivity = 0.58 (48/83)<br>Specificity = 0.79 (183/239)<br>PPV = 0.49 (48/99)<br>NPV = 0.84 (188/223)	**Moderate drop.**<br>AUC dropped from 0.88 to 0.76 (dro of 0.12).<br>Accuracy was comparable: internal 78% vs. external 77% (threshold 0.50) and 73% (threshold 0.24).<br><br>The authors state the model demonstrated "feasibility and generalizability" in the external cohort. However, they note "the current positive predictive values and sensitivity scores are not sufficient to withdraw predicted weak responders from DBS implantation," indicating the model should serve a "supportive and advisory role" rather than replacing clinical decision-making.<br><br>Overall: **moderate performance drop in discrimination (AUC) with maintained classification accuracy**. Model shows generalizability but with limitations in clinical utility for exclusion decisions.
Horn et al., 2017 [7]	Patient Selection / Outcome Prediction	STN DBS connectivity (structural and functional) from Parkinson's Disease patients. VTA-based connectivity profiles from normative connectome data (diffusion tractography and resting-state fMRI) used to predict UPDRS-III motor improvement. Training: 51 PD patients (Berlin). External test: 44 PD patients (Würzburg).	Berlin training dataset (n = 51): Functional connectivity: R = 0.44, p <0.001 (full); R = 0.40, p = 0.006 (leave-one-subcohort-out); Structural connectivity: R = 0.54, $p<10^{-7}$ (full); R = 0.46, p = 0.003 (leave-one-subcohort-out); Combined model (structural + functional): R^2 = 26.2%, R = 0.51, $p < 10^{-8}$; Combined + clinical variables: R^2 = 43.4%, R = 0.66, $p < 10^{-13}$; Leave-one-patient-out error: 15.7±14.2% (connectivity);	Würzburg test dataset (n = 44); Structural connectivity: R = 0.45, p = 0.002; Functional connectivity: R = 0.34, p = 0.03 (normative); R = 0.44, p < 0.005 (PD connectome) ; Structural (PD connectome): R = 0.38, p = 0.011	Functional: Internal R = 0.44 vs External R = 0.34 (drop of 0.10). Structural: Internal R = 0.54 vs External R = 0.45 (drop of 0.09). Both remained statistically significant in external cohort. **Reasonable generalizability despite differences in center, surgeon, outcome measure, and timing.** Authors note this is "the first DBS imaging study to test for reproducibility of results across independent and heterogeneous cohorts."

			15.1±11.8% (with clinical variables)		
Roediger et al., 2022 [8]	Programming	E-field-based predictive model (StimFit) for automated STN-DBS programming in Parkinson's Disease. Training: 612 monopolar review settings from 31 PD patients (University Hospital Cologne). Testing: 19 PD patients from independent center (Charité-Universitätsmedizin Berlin).	Motor improvement prediction (4-fold cross-validation): $R = 0.57$, $p < 10^{-10}$. Side-effect prediction: ROC AUC = 0.84. Median predicted side-effect probability: 9% (IQR: 2%–37%) without side effects vs. 76% (IQR: 43%–93%) with side effects ($p < 10^{-10}$).	Motor outcome difference prediction (independent test cohort, 19 patients): $R = 0.53$, $p < 10^{-3}$ (explained 28% of variance). Absolute outcome prediction across settings and patients: $R = 0.36$, $p < 10^{-3}$. StimFit setting agreement vs. clinical improvement (Dice coefficients): $R = 0.29$, $p = 0.041$. Side-effect prediction: Could not be evaluated (none of the chronic settings induced permanent side effects).	Motor prediction correlation **dropped modestly** from R = 0.57 (internal) to R = 0.53 (external, outcome differences) and R = 0.36 (external, absolute outcomes). Authors state: "Model performance within the training cohort was comparable to the performance in a cross-center independent test cohort, indicating generalizability of the model." Indirect StimFit validation via Dice coefficients showed only moderate correlation (R = 0.29). Side-effect model could not be externally validated.
Lange et al., 2023 [9]	Programming	GPi-DBS programming in isolated dystonia (generalised and cervical) using C-SURF algorithm based on probabilistic mapping. Model trained on 87 dystonia patients from 7 European centres. Prospectively tested on 10 new patients (8 analysed at follow-up). Motor outcomes measured via BFMDRS and TWSTRS.	Not reported in this paper. The underlying probabilistic map was built from 87 patients across seven centres and retrospectively predicted clinical improvements in unseen patients (reported in prior publications: Reich et al., 2019; Soares et al., 2021). Specific internal cross-validation metric values are not provided in this study.	Prospective testing on independent cohort (n = 8 analysed): **Dystonia symptom reduction:** 74.9 ± 15.3% with C-SURF vs. 66.3 ± 16.3% with clinical programming. **Statistical significance:** $P < 0.012$ (Wilcoxon signed-rank test); Cohen's d = 1.2. **Bayesian analysis:** Bayes Factor > 22.1; posterior δ = 1.37 (95% CI: 0.276–2.913). **Side-effect rate:** 5% (1/20 electrodes). 19/20 electrodes successfully programmed with C-SURF. **TEED:** 262.0 μJ/s (clinical) vs. 306.1 μJ/s (C-SURF) – not significantly different.	In this prospective external test, C-SURF was superior to expert clinical programming (74.9% vs. 66.3% symptom reduction, p < 0.012), suggesting robust generalisation of the probabilistic model to a new cohort. (<br>Authors note: "Overfitting of such models may be a critical limiting factor and could be mitigated if independent cohorts or prospective testing was considered."<br>Limitations: Small sample (n = 8 analysed), open-label feasibility design, short observation period (2 weeks + 4-month safety call). No worsening of symptom control or new-onset side effects at 4 months. 5/8 patients preferred C-SURF settings.
Sabo et al., 2023 [10]	Objective Automated Assessment	Vision-based (video) MDS-UPDRS-gait score prediction using ST-GCN model in adults with Parkinson's Disease	Not directly reported in this paper. The model was previously validated within the DIP dataset (ref [9]). Prior within-dataset	Macro-averaged Precision = 0.40 ± 0.12; Recall = 0.30 ± 0.05; F1-score = 0.22 ± 0.07; Unbalanced accuracy = 33.3%;	**Substantial performance drop.** F1-score dropped from 0.44-0.66 (internal) to 0.22 (external). Balanced accuracy dropped from 48-72% (internal) to 29.5%

		treated with STN-DBS and dopaminergic medication. Model trained on 362 videos from 14 older adults with drug-induced parkinsonism (DIP); externally tested on 42 videos from 13 individuals with PD.	performance: F1-scores of 0.44–0.66; balanced accuracies of 48–72%.	Balanced accuracy = 29.5%; Kendall's tau-b = 0.301 (p = 0.010); ICC with mean clinician scores = 0.19 (p = 0.112). Model detected ON/OFF treatment difference (p = 0.017, Cohen's d = 0.495).	(external). Authors state the model "struggled to transfer its ability to predict absolute MDS-UPDRS-gait scores across datasets." However, the model retained ability to detect relative within-individual changes in response to medication and DBS (p = 0.017), indicating partial robustness for change detection despite poor absolute score prediction.
Yin et al., 2024 [11]	Objective Automated Assessment	Basal ganglia LFP sleep stage decoding (Wake/NREM/REM) across multiple movement disorders (PD, Dystonia, HD, TS, ET) using the BGOOSE model (LightGBM classifier). Internal cohort: 114 patients from Beijing Tiantan Hospital. External cohorts: 12 PD-STN patients (Tsinghua, PINS device) and 3 PD patients (UCSF, Medtronic RC+S device).	Cross-all leave-one-subject-out cross-validation (n = 114): Accuracy = 85.8% (previously reported as ~85%); Balanced Accuracy = 77.6%; Weighted F1 score = 0.859 ; Per-stage accuracy: Wakefulness = 64.2%, NREM= 90.6%, REM = 71.8%; Average mislabeling rate = 8.85± 5.1 min per hour	Tsinghua dataset (n = 12 PD, PINS device): Accuracy = 78.3± 6.1% (range 66.7–86.7%); UCSF dataset (n = 3 PD, Medtronic RC+S): Accuracy = 79.8± 4.2% (range 66.7-86.7%) Combined external (n = 15); NREM accuracy = 81.7%, REM accuracy = 56.3%; Mislabeling rate = 12.8 ± 3.4 min per hour	**Moderate degradation.** Accuracy dropped from ~85.8% (internal LOOCV) to 78.3% (Tsinghua) and 79.8% (UCSF), representing a ~6-7.5% performance drop. The authors state BGOOSE "maintained around 80% accuracy during out-of-cohort validations." REM classification was notably poor in external data (56.3% vs 71.8% internally). Mislabeling rate increased from 8.85 to 12.8 min/hour. Performance drop may be attributed to differences in DBS devices, recording conditions (externalized vs chronically implanted), line noise frequency, and ECG artifact contamination. The model showed reasonable generalizability given differences in recording equipment and time points.
Peach et al., 2024 [12]	Objective Automated Assessment	Clinical video analysis of head movement dynamics in cervical dystonia (N = 86) and generalised dystonia (N = 30) patients, using visual perceptive deep learning (computer vision) to quantify disease states and DBS effects. Multicentre retrospective	Custom CNN (Model 2) for movement state classification: Training accuracy = 83.8%, Test accuracy = 84.6% (trained on 30 participants: 15 cervical dystonia, 15 healthy controls; tested on 15 participants: 8 cervical dystonia, 7 healthy controls). Head angle vs. clinical	The framework was applied to a fully independent, out-of-sample dataset of 30 generalised dystonia patients. The same five kinematic variables that exhibited the strongest response to DBS in the cervical dystonia cohort were also significantly modulated in the	No direct numeric comparison of identical metrics between internal and external cohorts is reported. The authors note: "we have applied our framework to diverse clinical videos from multiple centres with slightly differing protocols, showing the effects of neuromodulation in both focal and generalised dystonia, highlighting the generalisability of our tool and

		study across seven academic centres.	scores (Pearson r): Torticollis r = 0.79 (p = 5.6e-35), Laterocollis r = 0.67 (p = 1.2e-21), Anteroretrocollis r = 0.66 (p = 1.2e-18). Linear model predicting total TWSTRS severity score (combined kinematic + static features): MAE = 4.79. Oscillation amplitude vs. tremor score correlations: Torticollis r = 0.44 (p = 8.39e-9), Laterocollis r = 0.35 (p = 5.63e-6), Anteroretrocollis r = 0.39 (p = 2.32e-7).	generalised dystonia patients. Seven kinematic features significantly differentiated cervical from generalised dystonia subtypes. However, specific external validation metrics (e.g., accuracy, F1-score) on the independent cohort were not reported as numeric values comparable to internal testing.	specificity of our findings." The CNN test accuracy (84.6%) was marginally higher than training accuracy (83.8%), suggesting no overfitting. The independent generalised dystonia cohort confirmed the same top kinematic features were significantly modulated by DBS, indicating robustness across dystonia subtypes and centres. However, a formal quantitative performance drop analysis was not reported.
Friedrich et al., 2024 [13]	Objective Automated Assessment	Computer vision (Mediapipe) tremor analysis from clinical video recordings of Essential Tremor patients with thalamic DBS. Prospective cohort (n = 8) used for validation; independent retrospective cohort (n = 43 postural, n = 25 kinetic) from two clinical sites used for external clinical validation.	Prospective validation cohort: Postural tremor amplitude: Spearman's ρ = 0.86 (vs. clinical scores, p < 0.001); ρ = 0.89 (vs. motion capture, p < 0.001); MAE = 10 mm (95% CI [5.65, 14.4]). Kinetic tremor amplitude: ρ = 0.55 (vs. scores, p < 0.01); ρ = 0.72 (vs. motion capture, p < 0.001); MAE = −2.60 mm (95% CI [−3.13, 8.23]). Postural tremor frequency: MAE = −0.21 Hz (95% CI [−0.05, 0.46]). Kinetic tremor frequency: MAE = −0.06 Hz (95% CI [−0.30, 0.41]). DBS outcome prediction (logistic regression, LOOCV): AUROC = 0.88, F1-score = 0.89, balanced accuracy = 0.88.	Independent retrospective cohort (external clinical validation): Postural tremor amplitude: Spearman's ρ = 0.65 (vs. clinical scores, p < 0.001); DBS responsiveness: r = 0.49 (95% CI [0.34, 0.61], p < 0.001). Kinetic tremor amplitude: ρ = 0.42 (vs. scores, p < 0.001); DBS responsiveness: r = 0.37 (95% CI [0.03, 0.61], p = 0.025). Postural tremor frequency: 5.96 ± 0.76 Hz. Kinetic tremor frequency: 5.75 ± 0.58 Hz.	Postural tremor amplitude score correlation dropped from ρ = 0.86 (prospective) to ρ = 0.65 (retrospective), a decrease of 0.21. Kinetic tremor amplitude score correlation dropped from ρ = 0.55 to ρ = 0.42, a decrease of 0.13. The framework remained responsive to DBS effects in both cohorts. The retrospective cohort used unstandardised, real-world legacy videos from two different clinical sites with varying camera setups, which likely accounts for the performance reduction. Authors note the framework's "generalisability and versatility" despite these challenges. No gold-standard motion capture comparison was available in the retrospective cohort.
Kerman et al., 2022 [14]	Adaptive DBS	Beta oscillation detection in STN for Parkinson's Disease. **Training data:** PD computational model (cortex-basal ganglia-thalamus-cortex circuit;	**Training Accuracy:** MLP = 0.99; SNN = 0.93<br>**Inference Accuracy:** MLP = 0.7850; SNN = 0.715	**Not reported.** The paper states the detector was "evaluated using experimental data from the PD rats," but no specific classification performance	**Not reported.** No direct comparison of classification performance between computational model data (internal) and experimental rat data (external) is provided. The

		10,000 samples).<br>**Testing data:**<br>Experimental data from four 6-OHDA lesioned rats using optogenetic technique.		metrics (accuracy, F1, AUC, etc.) are provided for the external experimental rat data. Only hardware performance metrics are reported for the memristive implementation (chip area -67.3%, latency -41.9%, energy -11.7% vs SRAM).	paper notes experimental data had “higher power spectrum values than the training samples” requiring scaling factors for alignment, suggesting a domain gap. No discussion of generalization, overfitting, or performance drop.

References:

1. Boutet, A. *et al.* Predicting optimal deep brain stimulation parameters for Parkinson’s disease using functional MRI and machine learning. *Nat Commun* **12**, 3043 (2021).
2. Boutet, A. *et al.* Sign-specific stimulation ‘hot’ and ‘cold’ spots in Parkinson’s disease validated with machine learning. *Brain Communications* **3**, fcab027 (2021).
3. Shamir, R. R. *et al.* Microelectrode recordings validate the clinical visualization of subthalamic-nucleus based on 7T magnetic resonance imaging and machine learning for deep brain stimulation surgery. *Neurosurg.* **84**, 749–757 (2019).
4. Valsky, D. *et al.* Real-time machine learning classification of pallidal borders during deep brain stimulation surgery. *J. Neural Eng.* **17**, 016021 (2020).
5. Chang, B. *et al.* Comprehensive clinical scale-based machine learning model for predicting subthalamic nucleus deep brain stimulation outcomes in Parkinson’s disease. *Neurosurg Rev* **48**, 266 (2025).
6. Habets, J. G. V. *et al.* Multicenter Validation of Individual Preoperative Motor Outcome Prediction for Deep Brain Stimulation in Parkinson’s Disease. *Stereotact Funct Neurosurg* **100**, 121–129 (2022).
7. Horn, A. *et al.* Connectivity Predicts deep brain stimulation outcome in P arkinson disease. *Annals of Neurology* **82**, 67–78 (2017).
8. Roediger, J. *et al.* StimFit— A Data-Driven Algorithm for Automated Deep Brain Stimulation Programming. *Movement Disorders* **37**, 574–584 (2022).
9. Lange, F. *et al.* Machine versus physician-based programming of deep brain stimulation in isolated dystonia: A feasibility study. *Brain Stimul.* **16**, 1105–1111 (2023).
10. Sabo, A., Iaboni, A., Taati, B., Fasano, A. & Gorodetsky, C. Evaluating the ability of a predictive vision-based machine learning model to measure changes in gait in response to medication and DBS within individuals with Parkinson’s disease. *BioMed. Eng. OnLine* **22**, 120 (2023).
11. Yin, Z. *et al.* Generalized sleep decoding with basal ganglia signals in multiple movement disorders. *npj Digit. Med.* **7**, 122 (2024).
12. Peach, R. *et al.* Head movement dynamics in dystonia: a multi-centre retrospective study using visual perceptive deep learning. *npj Digit. Med.* **7**, 160 (2024).
13. Friedrich, M. U. *et al.* Validation and application of computer vision algorithms for video-based tremor analysis. *npj Digit. Med.* **7**, 165 (2024).

14. Kerman, Z., Yu, C. & An, H. Beta Oscillation Detector Design for Closed-Loop Deep Brain Stimulation of Parkinson's Disease with Memristive Spiking Neural Networks. in *2022 23rd International Symposium on Quality Electronic Design (ISQED)* 1–6 (IEEE, Santa Clara, CA, USA, 2022). doi:10.1109/ISQED54688.2022.9806207.

PRISMA

PRISMA 2020 Checklist

Section and Topic	Item #	Checklist item	Location where item is reported
**TITLE**			
Title	1	Identify the report as a systematic review.	Title
**ABSTRACT**			
Abstract	2	See the PRISMA 2020 for Abstracts checklist.	Abstract
**INTRODUCTION**			
Rationale	3	Describe the rationale for the review in the context of existing knowledge.	Introduction: paragraphs 1-4.
Objectives	4	Provide an explicit statement of the objective(s) or question(s) the review addresses.	Introduction: final paragraph (four enumerated questions); primary question stated in the preceding paragraph.
**METHODS**			
Eligibility criteria	5	Specify the inclusion and exclusion criteria for the review and how studies were grouped for the syntheses.	Methods: 'Eligibility Criteria', 'Data Extraction and Synthesis', paragraph 2.
Information sources	6	Specify all databases, registers, websites, organisations, reference lists and other sources searched or consulted to identify studies. Specify the date when each source was last searched or consulted.	Methods: 'Search strategy and selection criteria', paragraph 1
Search strategy	7	Present the full search strategies for all databases, registers and websites, including any filters and limits used.	Methods: 'Search strategy and selection criteria', paragraph 1; full strategies in the Figshare repository (doi:10.6084/m9.figshare.31750372).
Selection process	8	Specify the methods used to decide whether a study met the inclusion criteria of the review, including how many reviewers screened each record and each report retrieved, whether they worked independently, and if applicable, details of automation tools used in the process.	Methods: 'Search strategy and selection criteria', paragraph 1
Data collection process	9	Specify the methods used to collect data from reports, including how many reviewers collected data from each report, whether they worked independently, any processes for obtaining or confirming data from study investigators, and if applicable, details of automation tools used in the process.	Methods: 'Data Extraction and Synthesis', paragraph 1; dual-reviewer procedure cross-referenced in 'Search strategy and selection criteria'
Data items	10a	List and define all outcomes for which data were sought. Specify whether all results that were compatible with each outcome domain in each study were sought (e.g. for all measures, time points, analyses), and if not, the methods used to decide which results to collect.	Methods: 'Data Extraction and Synthesis', paragraph 1; performance-metric and validation definitions in the same section.
	10b	List and define all other variables for which data were sought (e.g. participant and intervention characteristics, funding sources). Describe any assumptions made about any missing or unclear information.	Methods: 'Data Extraction and Synthesis', paragraph 1 (seven enumerated variable groups plus governance/deployment variables).
Study risk of bias assessment	11	Specify the methods used to assess risk of bias in the included studies, including details of the tool(s) used, how many reviewers assessed each study and whether they worked independently, and if applicable, details of automation tools used in the process.	Methods: 'Risk of bias and reporting quality', paragraphs 1-2.
Effect measures	12	Specify for each outcome the effect measure(s) (e.g. risk ratio, mean difference) used in the synthesis or presentation of results.	NA
Synthesis	13a	Describe the processes used to decide which studies were eligible for each synthesis (e.g. tabulating the study	Methods: 'Data Extraction and

Section and Topic	Item #	Checklist item	Location where item is reported
methods		intervention characteristics and comparing against the planned groups for each synthesis (item #5)).	Synthesis', paragraph 2.
	13b	Describe any methods required to prepare the data for presentation or synthesis, such as handling of missing summary statistics, or data conversions.	NA
	13c	Describe any methods used to tabulate or visually display results of individual studies and syntheses.	Methods: 'Data Extraction and Synthesis', paragraph 2; Results (Tables 1-4; Figures 2-5).
	13d	Describe any methods used to synthesize results and provide a rationale for the choice(s). If meta-analysis was performed, describe the model(s), method(s) to identify the presence and extent of statistical heterogeneity, and software package(s) used.	Methods: 'Data Extraction and Synthesis', paragraphs 1-2.
	13e	Describe any methods used to explore possible causes of heterogeneity among study results (e.g. subgroup analysis, meta-regression).	NA
	13f	Describe any sensitivity analyses conducted to assess robustness of the synthesized results.	NA
Reporting bias assessment	14	Describe any methods used to assess risk of bias due to missing results in a synthesis (arising from reporting biases).	Methods: 'Risk of bias and reporting quality'
Certainty assessment	15	Describe any methods used to assess certainty (or confidence) in the body of evidence for an outcome.	NA
**RESULTS**			
Study selection	16a	Describe the results of the search and selection process, from the number of records identified in the search to the number of studies included in the review, ideally using a flow diagram.	Results: 'Study characteristics', paragraph 1; Figure 1 (PRISMA flow diagram).
	16b	Cite studies that might appear to meet the inclusion criteria, but which were excluded, and explain why they were excluded.	NA
Study characteristics	17	Cite each included study and present its characteristics.	Results: 'Study characteristics'; Table 1; full per-study list on Figshare (doi:10.6084/m9.figshare.31750372).
Risk of bias in studies	18	Present assessments of risk of bias for each included study.	NA
Results of individual studies	19	For all outcomes, present, for each study: (a) summary statistics for each group (where appropriate) and (b) an effect estimate and its precision (e.g. confidence/credible interval), ideally using structured tables or plots.	Table 1; full per-study list on Figshare (doi:10.6084/m9.figshare.31750372).
Results of syntheses	20a	For each synthesis, briefly summarise the characteristics and risk of bias among contributing studies.	NA
	20b	Present results of all statistical syntheses conducted. If meta-analysis was done, present for each the summary estimate and its precision (e.g. confidence/credible interval) and measures of statistical heterogeneity. If comparing groups, describe the direction of the effect.	NA
	20c	Present results of all investigations of possible causes of heterogeneity among study results.	NA
	20d	Present results of all sensitivity analyses conducted to assess the robustness of the synthesized results.	NA
Reporting biases	21	Present assessments of risk of bias due to missing results (arising from reporting biases) for each synthesis assessed.	NA
Certainty of evidence	22	Present assessments of certainty (or confidence) in the body of evidence for each outcome assessed.	NA

PRISMA 2020 Checklist

Section and Topic	Item #	Checklist item	Location where item is reported
**DISCUSSION**			
Discussion	23a	Provide a general interpretation of the results in the context of other evidence.	Discussion: paragraphs 1-12
	23b	Discuss any limitations of the evidence included in the review.	Discussion: paragraphs discussing study-design characteristics and metric/calibration limitations; dedicated limitations paragraph.
	23c	Discuss any limitations of the review processes used.	Discussion: final two limitations paragraphs (“Scope was restricted…” and the TRL-framework paragraph).
	23d	Discuss implications of the results for practice, policy, and future research.	Discussion: reform/recommendation paragraphs (validation designs, reporting standards, governance, federated learning, benchmarks, funding, foundation models).
**OTHER INFORMATION**			
Registration and protocol	24a	Provide registration information for the review, including register name and registration number, or state that the review was not registered.	Methods: First paragraph.
	24b	Indicate where the review protocol can be accessed, or state that a protocol was not prepared.	Methods: First paragraph (PROSPERO registration).
	24c	Describe and explain any amendments to information provided at registration or in the protocol.	Methods: First paragraph (second sentence).
Support	25	Describe sources of financial or non-financial support for the review, and the role of the funders or sponsors in the review.	‘Acknowledgements’ section.
Competing interests	26	Declare any competing interests of review authors.	‘Competing interest’ section.
Availability of data, code and other materials	27	Report which of the following are publicly available and where they can be found: template data collection forms; data extracted from included studies; data used for all analyses; analytic code; any other materials used in the review.	‘Data availability’ section; also cited in Methods and Results (Figshare doi:10.6084/m9.figshare.31750372).

From: Page MJ, McKenzie JE, Bossuyt PM, Boutron I, Hoffmann TC, Mulrow CD, et al. The PRISMA 2020 statement: an updated guideline for reporting systematic reviews. BMJ 2021;372:n71. doi: 10.1136/bmj.n71. This work is licensed under CC BY 4.0. To view a copy of this license, visit https://creativecommons.org/licenses/by/4.0/
